# A pH- and Bioreducible Cationic Copolymer with Amino Acids and Piperazines for Adenovirus Delivery

**DOI:** 10.3390/pharmaceutics14030597

**Published:** 2022-03-09

**Authors:** Thavasyappan Thambi, Jeongmin Lee, A-Rum Yoon, Dayananda Kasala, Chae-Ok Yun

**Affiliations:** 1 Department of Bioengineering, College of Engineering, Hanyang University, 222 Wangsimni-ro Seongdong-gu, Seoul 04763, Korea; thambi1983@gmail.com (T.T.); skymjn@daum.net (J.L.); ayoon@hanyang.ac.kr (A.-R.Y.); kasala@hanyang.ac.kr (D.K.); 2 GeneMedicine Co., Ltd., 222 Wangsimni-ro Seongdong-gu, Seoul 04763, Korea; 3 Hanyang Institute of Bioscience and Biotechnology (HY-IBB), Hanyang University, Seoul 04763, Korea; 4 Institute of Nano Science and Technology (INST), Hanyang University, 222 Wangsimni-ro, Seongdong-gu, Seoul 04763, Korea

**Keywords:** adenovirus, systemic delivery, nonimmunogenic polymer, tumor-targeted delivery, cancer gene therapy

## Abstract

Adenoviruses (Ads) are attractive nonviral vectors and show great potential in cancer gene therapy. However, inherent properties of Ads, including immunogenicity, nonspecific toxicity, and coxsackie and adenovirus receptor (CAR)-dependent cell uptake, limit their clinical use. To surmount these issues, we developed a pH- and glutathione-responsive poly(ethylene glycol)-poly(ꞵ-aminoester)-polyethyleneimine (PPA) for conjugation with Ad. The pH sensitivity of the PPA copolymer was elegantly tuned by substitution with different amino acids (arginine, histidine, and tryptophan), piperazines (Pip1, Pip2, and Pip3), and guanidine residues in the backbone of the PPA conjugate. PPA copolymer was further functionalized with short-chain cross-linker succinimidyl 3-(2-pyridyldithio)propionate) (SPDP) to obtain PPA-SPDP for facile conjugation with Ad. The PPA-conjugated Ad (PPA-Ad) conjugate was obtained by reacting PPA-SPDP conjugate with thiolated Ad (Ad-SH). Ad-SH was prepared by reacting Ad with 2-iminothiolane. The size distribution and zeta potential results of PPA-Ad conjugate showed an increasing trend with an increase in copolymer dose. From in vitro test, it was found that the transduction efficiency of PPA-Ad conjugate in CAR-positive cells (A549 and H460 cells) was remarkably increased at the acidic pH condition (pH 6.2) when compared with PPA-Ad conjugate incubated under the physiological condition (pH 7.4). Interestingly, the increase in transduction efficiency was evidenced in CAR-negative cells (MDA-MB-231 and T24 cells). These results demonstrated that biocompatible and biodegradable PPA copolymers can efficiently cover the surface of Ad and can increase the transduction efficiency, and hence PPA copolymers can be a useful nanomaterial for viral vector delivery in cancer therapy.

## 1. Introduction

Gene therapy has the potential to cure a wide range of genetically inherited diseases [1]. For successful gene therapy, safe and efficient gene delivery vectors are important to overcome certain safety issues, including immunogenicity and toxicity [2,3,4,5]. In recent years, viral vectors have received great attention as potential delivery vectors for gene therapy [6,7,8]. Thus far, numerous viral vectors, including lentivirus, adenovirus (Ad), adeno-associated virus, and retrovirus, have often been used as viral gene delivery systems [5,9]. Among them, Ad is a promising candidate and extensively investigated in viral-based gene therapy owing to its high gene delivery efficacy, no risk of insertional mutagenesis, and facile production of viral vectors at high titers [10,11,12]. Owing to these features, viral gene delivery vectors received attention in gene delivery [13].

Ad can bind tumor cells specifically by binding to its overexpressed receptors [14]. In general, Ads enter cells through two-step processes. Firstly, binding of Ad fiber protein to coxsackie and Ad receptor (CAR) occurs; the binding is subsequently assisted by affinity interaction between cellular integrins (α*_v_*β*_3_* and α*_v_*β*_5_*) and arginine–glycine–aspartate (RGD) motifs located on the penton bases of Ad [15,16,17,18]. Importantly, high expression levels of CAR in various cell types have been reported to cause nonspecific distribution of Ads in vivo [19]. Furthermore, large variability in the CAR expression levels of heterogenic clinical tumors can prevent effective infection by oAd [20,21], thus resulting in suboptimal therapeutic efficacy. Due to these reasons, Ad-mediated gene delivery is severely hammered. To minimize limitations and improve gene delivery efficiency without sacrificing their biological properties, nonviral carriers such as cationic copolymers are explored [22,23,24,25].

Cationic polymers are an important class of nonviral vectors because of their tunable molecular weight and morphology, chemical structure, and adjustability to control the cationic characteristics to bind with anionic biotherapeutics, including Ad [26,27,28]. Among various cationic polymers, natural polysaccharides, such as chitosan [29], and synthetic polymers poly(ethylene imine) (PEI) [30], poly(L-lysine) (PLL) [31], and poly(amidoamine) (PAMAM) [32] have often been used for delivery of anionic biotherapeutics. Nevertheless, most of these cationic polymers are nondegradable or degrade slowly through hydrolytic degradation in a sustained manner [33,34]. In addition, long-term use of nondegradable polymers leads to vacuolization [35]. Hence, cationic polymers that degrade in a short period of time in the body remain a major target [36]. Such cationic-polymer-covered Ad vectors allow effective binding of the complex on the surface of cancer cells [37]. However, most of the cationic polymers used to coat Ad vectors are nondegradable with limited biocompatibility [38]. Ads coated or conjugated with nondegradable cationic polymers or neutral polymers often exhibit poor transduction because of the delayed release into cancer cells [39]. Therefore, to overcome nondegradability and trigger the release of Ad into the cancer cells, a cationic polymer with biodegradable characteristics with effective condensation ability or reaction functional sites for conjugation is important.

Herein, we developed a series of pH- and reduction-responsive poly(ethylene glycol)-poly(ꞵ-aminoester)-polyethyleneimine (PPA) copolymer finely grafted with amino acids (arginine (Arg), histidine (His), and tryptophan (Trp)), piperazines (P1, P2, and P3), and guanidine. The presence of stimuli-responsive functional moieties in the PPA copolymers reduces cytotoxicity and improves transduction efficiency. The PPA copolymers were conjugated with succinimidyl 3-(2-pyridyldithio)propionate (SPDP) (PPA-SPDP) to chemically conjugate on the surface of thiolated-Ad (Ad-SH) through the thiol exchange reaction. The optimal concentration to cover the surface of Ad by copolymer was investigated by varying the molar ratio of PPA copolymers to a fixed amount of Ad (1 × 10^10^ vp) using size and zeta potential measurement. In vitro cytotoxicity and transduction efficiency of PPA copolymers were examined using CAR-positive and CAR-negative cell lines.

## 2. Materials and Methods

### 2.1. Chemicals

Poly(ethylene glycol) monomethyl ether monoacrylate (mPEG-Ac, M_n_ = 5000 g/mol) was obtained from Lasan Bio Inc. Electro Pure *N,N’*-cystaminebisacrylamide (CBA) was supplied by PolySciences Inc. SPDP was purchased from Tokyo Chemical Industries (Tokyo, Japan). Piperazine, poly(ethyleneimine) solution 50 wt.% in water (PEI, M_n_ = 1800 g/mol), *N*-hydroxysuccinimide (NHS), l-arginine (Arg), l-histidine (His), l-tryptophan (Trp), 1-ethyl-3-(3′-dimethylaminopropyl)carbodiimide·HCl (EDC), piperazine-2-carboxylic acid dihydrochloride (P1), 1-amino-4-(2-hydroxyethyl)piperazine (P2), 3-(4-methyl-1-piperazinyl)propanoic acid dihydrochloride (P3), 3-(4,5-dimethylthiazol-2-yl)-2,5-diphenyl-tetrazolium bromide (MTT), 1H-pyrazole-1-carboxamidine hydrochloride (Gua), and 2-iminothiolane hydrochloride (2-IT) were supplied by Sigma Aldrich.

### 2.2. Cancer Cells and Ad Preparations

Human lung cancer cell lines (A549 and H460), urinary bladder cancer cell line (T24), human breast cancer cell line (MDA-MB-231), and Ad E1A-expressing HEK293 cells were purchased from the American Type Culture Collection (ATCC, Manassas, VA, USA). The cells were cultured in Dulbecco’s Modified Eagle’s Medium (DMEM) or RPMI 1640 medium containing 10% fetal bovine serum (FBS) at 37 °C in an incubator with 5% CO_2_. The replication-incompetent Ad expressing green fluorescent protein (GFP) (dE1/GFP) was prepared according to our previous report [40]. Hereafter, the dE1/GFP is referred to as Ad. In brief, E1-deleted green fluorescence protein expressing Ad (dE1-GFP) was propagated in HEK293 cells and purified using a cesium chloride gradient. Viral particle (VP) number was calculated by measuring the optical density at 260 nm (OD_260_), for which an absorbance value of 1 was equivalent to 1 × 1.1 × 10^12^ VP/mL.

### 2.3. Synthesis of PPA Copolymers

**Scheme 1:**
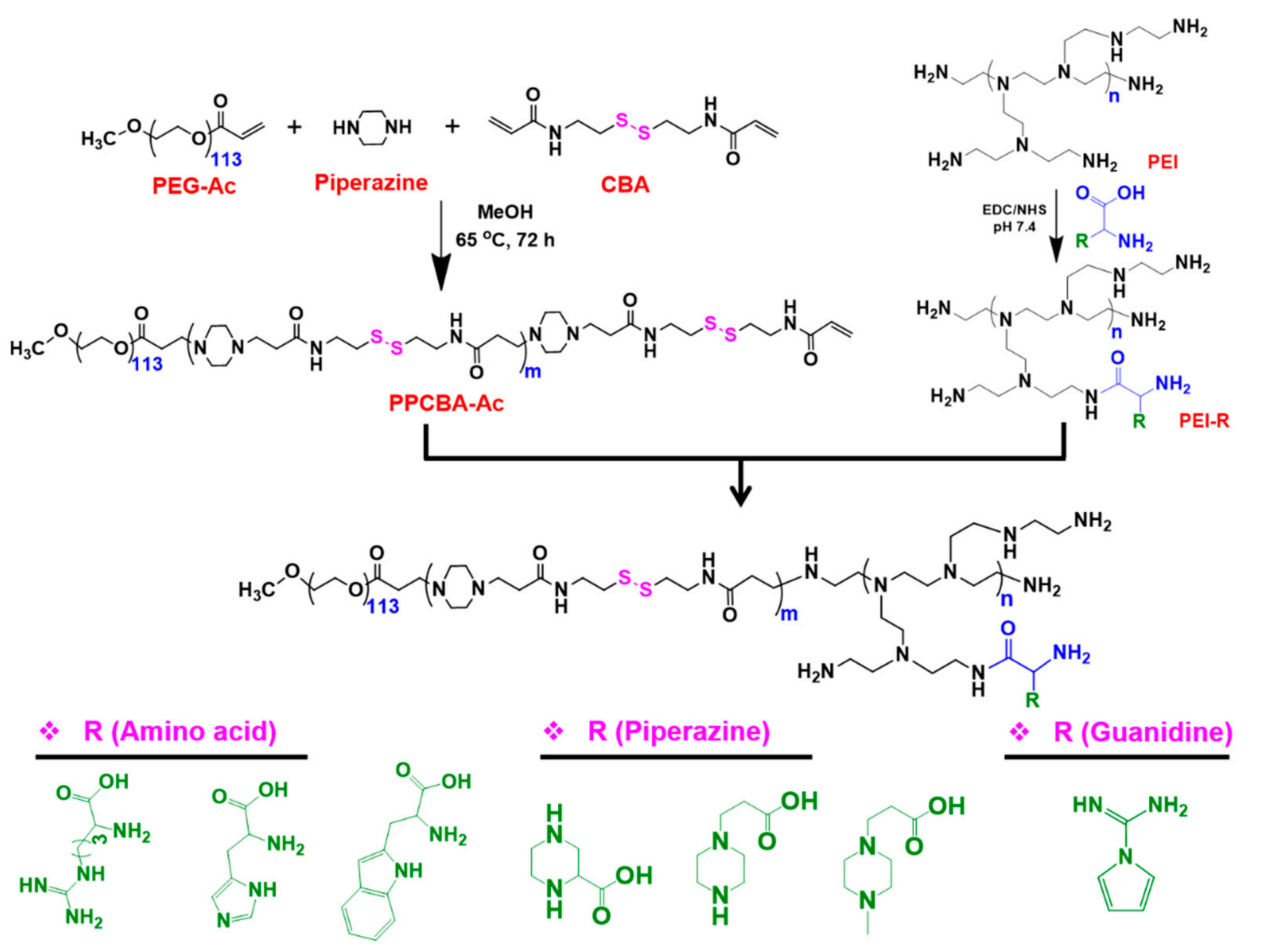
Synthesis of PPA copolymers. The different substituents including amino acids, piperazines, and guanidine are used for conjugation with PEI.

A library of PPA-SPDP conjugates was synthesized using a combinations of individually synthesized poly(ethylene glycol)-poly(ꞵ-aminoester) (PPCBA) copolymers and PEI conjugates (Scheme 1). Firstly, PPCBA copolymer was synthesized by the Michael addition polymerization reaction [41]. Secondly, PEIs conjugated with seven different functional moieties with different water solubilities and pH sensitivities were synthesized. Finally, PPCBA copolymer and PEI conjugate were combined by Michael addition reaction to produce PPA conjugate.

### 2.4. Synthesis of Acrylate-Terminated Copolymer (PPCBA)

PEG-acrylate (200 mg, 1 eq), CBA (14 eq), and amine (10 eq) were taken in a round-bottom (RB) flask. Methanol (5 mL) was added and stirred for 15 min to obtain a clear solution. Thereafter, the RB flask was kept in a prewarmed oil bath (60 °C) and stirred at the same temperature under nitrogen for 72 h. The crude copolymer was then cooled to 0 °C, and excess diethyl ether (6- to 10-fold) was added to obtain the precipitate. Then, the precipitate was filtered and dried under vacuum. The precipitate was dispersed in DIW and passed through a 0.45 µm cellulose acetate filter. Finally, the filtrate was frozen and lyophilized to obtain PPCBA-Ac product.

### 2.5. Synthesis of PEI Conjugates

Arg, His, Trp, P1, P2, or P3 (1 eq) was dissolved in PBS (pH 7.4, 10 mg/mL concentration) followed by the addition of NHS (10 eq). Thereafter, the reaction mixture was cooled to 0 °C and EDC (10 eq) was added, and the stirring continued for 3 h. PEI (1 g) was then added, and the reaction continued at room temperature for 18 h. The reaction mixture was then dialyzed against DIW (MWCO: 1000 Da), and the dialysate was passed through a 0.45 µm cellulose acetate filter. The clear solution was frozen and lyophilized to obtain PEI conjugates. The obtained conjugates were coded based on the different functional moieties (PEI-Arg, PEI-His, PEI-Trp, PEI-P1, PEI-P2, and PEI-P3). The PEI-Gua conjugate was synthesized by simple mixing of Gua and PEI in PBS. The purification of PEI-Gua conjugate was done similarly to that of other PEI conjugates.

### 2.6. Synthesis of PPA Copolymers

PPCBA copolymer (1 eq) and PEI-Arg conjugate (3 eq) were taken in a round-bottom (RB) flask. Methanol/water (5 mL; 4v/1v) was added and stirred for 15 min to obtain a clear solution. Thereafter, the RB flask was kept in a prewarmed oil bath (60 °C) and stirred for 3 days. The crude product was then dialyzed against water (MWCO: 3500 Da), and the dialysate was passed through a 0.45 µm cellulose acetate filter. The clear solution was frozen and lyophilized to obtain PPA copolymers. Similarly, other PEI conjugates (PEI-His, PEI-Trp, PEI-P1, PEI-P2, PEI-P3, and PEI-Gua) were reacted with PPCBA copolymer. The conjugates were coded depending on the substituents in the PEI backbone. For example, PPH indicates that PEI conjugate possesses histidine residues.

### 2.7. Synthesis of PPA-SPDP Copolymers

PPA copolymer (1 eq) was dispersed in PBS buffer and stirred at 0 °C. SPDP in DMF was added slowly, and the reaction was continued for 3 h at room temperature. The crude product was then dialyzed against water (MWCO: 3500 Da), and the dialysate was passed through a 0.45 µm cellulose acetate filter. The clear solution was frozen and lyophilized to obtain PPA-SPDP copolymers.

### 2.8. Preparation of PPA-SPDP Copolymer Conjugated Ad (PPA-Ad)

The PPA-SPDP copolymer was conjugated on the surface of Ads via thiol exchange reaction. Firstly, thiolated Ad (Ad-SH) was obtained by treating Ad using 2-IT. Thereafter, the Ad-SH was reacted with PPA-SPDP copolymer via the disulfide exchange reaction to obtain PPA-Ad conjugates.

### 2.9. Characterization

#### 2.9.1. ^1^H NMR Spectra

The intermediate copolymer/conjugates and final PPA copolymers were characterized using Varian 600 MHz NMR instrument (VARIAN 600 MHz; Varian Inc., Palo Alto, CA, USA).

#### 2.9.2. Size and Zeta Potential

The size and zeta potential of PPA-Ad conjugates were measured with a Zetasizer 3000HS (Malvern Instruments Inc., Worcestershire, UK). To estimate the optimal copolymer amount, various concentrations of PPA-SPDP copolymer (2 × 10^4^, 5 × 10^4^, 1 × 10^5^, and 2 × 10^5^) were gently mixed with Ad-SH (5 × 10^9^ vp) and incubated for 30 min. After the preparation of PPA-Ad conjugate, phosphate-buffered saline was added to a final volume of 1.0 mL and adjusted to the desired pH before analysis.

#### 2.9.3. Transmission Electron Microscopy (TEM)

The morphologies of fresh Ad and PPA-Ad conjugate were investigated by TEM (JEM-2000EXll, JEPL). Fresh Ad (5 × 10^9^ VP) and PPA-SPDP copolymer (1 × 10^4^, 2 × 10^4^, 5 × 10^4^, 1 × 10^5^, or 2 × 10^5^ with 5 × 10^9^ VP) were placed on copper grids. The solution was removed using filter paper and stained using 1% aqueous uranyl acetate. The staining solution was removed and the Ad-loaded grid was dried. Finally, images were obtained using TEM at 200 kV.

#### 2.9.4. Acid–Base Titration

The pH-buffering capacity and pH sensitivity of the PPA copolymers were measured by acid–base titration [42]. For the acid–base titration test, 25 mg of copolymers were dispersed in PBS and the pH was adjusted to 3 using 0.1N HCl. Thereafter, the pH of the copolymer solution was gradually raised by adding 50 µL, and pH was measured with a pH meter. Finally, the titration was obtained by plotting against change in pH versus added volume of 0.1N NaOH. pKa value of the copolymer was determined from the titration using a geometric method.

### 2.10. Gel Retardation Assay

To investigate the effective Ad covering ability, various PPA-Ad conjugates containing different weight ratios of PPA were prepared. The PPA-Ad conjugates were treated with virus lysis buffer and subsequently incubated for 30 min at 56 °C. Thereafter, the lysed samples were loaded onto a 0.8% (*w*/*v*) agarose gel in 1X TAE buffer containing ethidium bromide. The sample-loaded gels were electrophoresed for 30 min at 120 V. DNA bands were finally visualized using a ChemiDoc.

### 2.11. MTT Assay

To evaluate the cancer-cell-killing effect, A549 cells, H460 cells, MDA-MB-231 cells, and T24 cells were grown in 24-well plates (~50% confluence). The cells were then exposed to various PPA copolymers. After 3 days, MTT (250 µL, 2 mg/mL) in PBS was added and incubated for 3 h. The purple formazan product was dissolved in DMSO, and the optical density was recorded on a microplate reader at 540 nm. The cells exposed to only culture medium served as negative controls.

### 2.12. Transduction of PPA-Ad Conjugate in Cancer Cells

Transduction efficiency of PPA-Ad conjugate was analyzed by quantifying GFP expression using Incucyte Live-Cell Analysis in CAR-positive (A549 cells and H460 cells) and CAR-negative (MDA-MB-231 cells and T24 cells) cell lines. Cells were seeded in 48-well plates at a density of 1 × 10^4^ cells/well for 24 h. Thereafter, cells were infected with naked Ad or PPA-Ad conjugate (1 × 10^4^, 2 × 10^4^, 5 × 10^4^, 1 × 10^5^, or 2 × 10^5^) with 15 MOI in a serum-free condition at different pH conditions (pH 7.4 and pH 6.0) at 37 °C for 4 h. The culture medium was then removed and exchanged with 5% FBS containing fresh medium. Finally, cells were photographed under a fluorescence microscope (Olympus IX81; Olympus Optical, Tokyo, Japan) after 2 days. The GFP expression was quantified using FACS analysis BD FACScan analyzer (Beckton-Dickinson, CA, USA).

### 2.13. Statistical Analysis

The results are presented as mean ± standard deviation. Statistical analysis between Ad and sample groups was performed using the Student *t*-test. Sample groups with *p* < 0.05 were considered statistically significant.

## 3. Results and Discussion

**Scheme 2:**
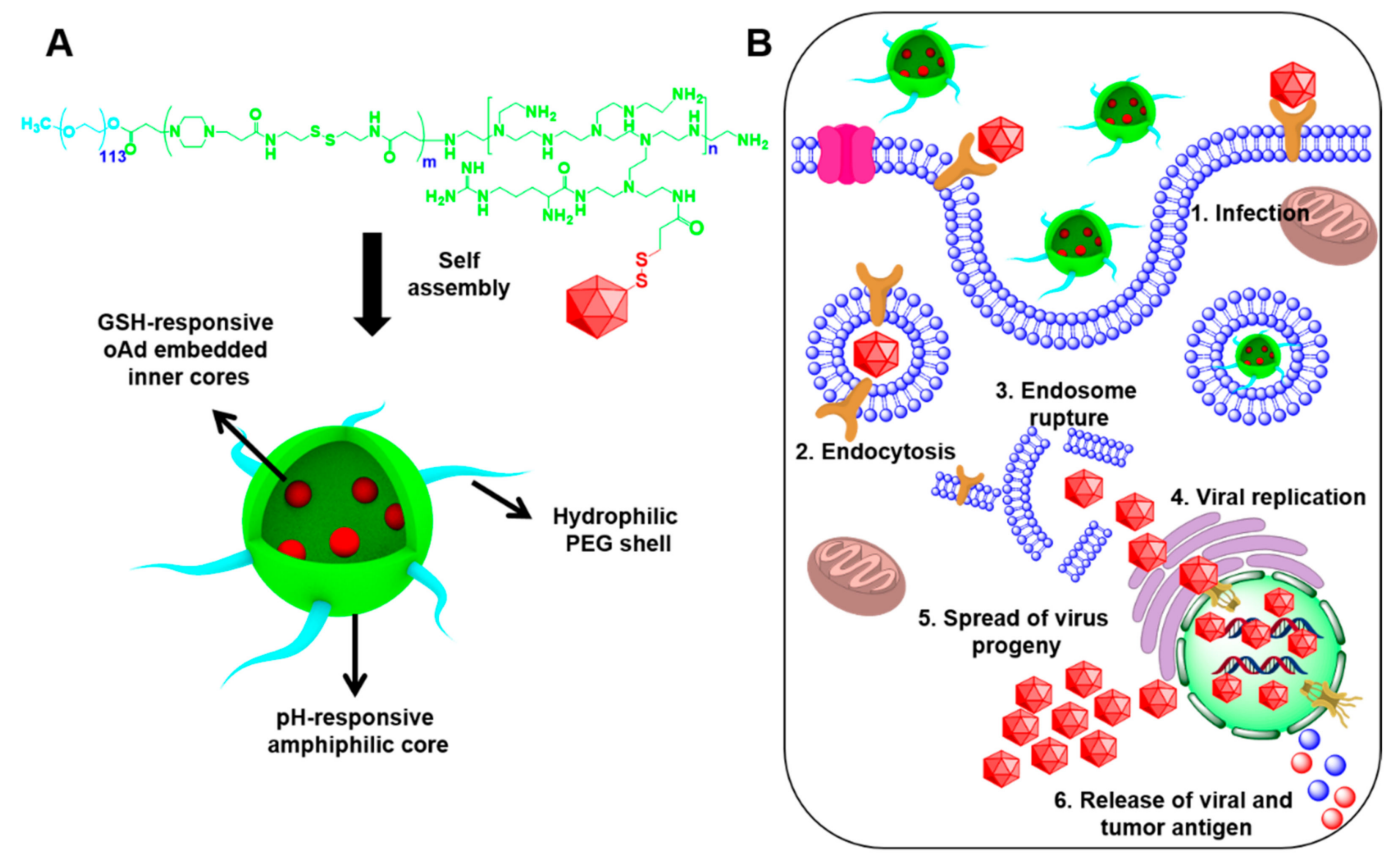
Schematic illustration of (**A**) self-assembly of Ad-conjugated PPA copolymers in aqueous solutions and (**B**) internalization of cationic PPA-Ad conjugates by binding to the anionic membrane of cancer cells and subsequent release of Ad by a sequential three-step process.

In recent years, Ad-based nonviral vectors have emerged as promising vehicles for cancer therapy applications [5,43]. To improve the internalization of negatively charged Ad vectors, the surface of Ad capsids is often complexed with cationic polymers that can assist adhesion to the negatively charged cell membrane and enhance the internalization of Ad vectors [44]. Since the Ad vectors are covered using cationic polymers, the viral uptake is enhanced regardless of CAR expression level [45]. The surface-covered PPA-Ad conjugate is effectively internalized into the cells by ionic interaction, and the presence of GSH in the cell cytoplasm reduces the disulfide bonds and triggers the release of Ad (Scheme 2).

### 3.1. Synthesis and Characterization of PPA Copolymers

The PPA copolymers were prepared using a two-step process as presented in Scheme 1.

**Figure 1:**
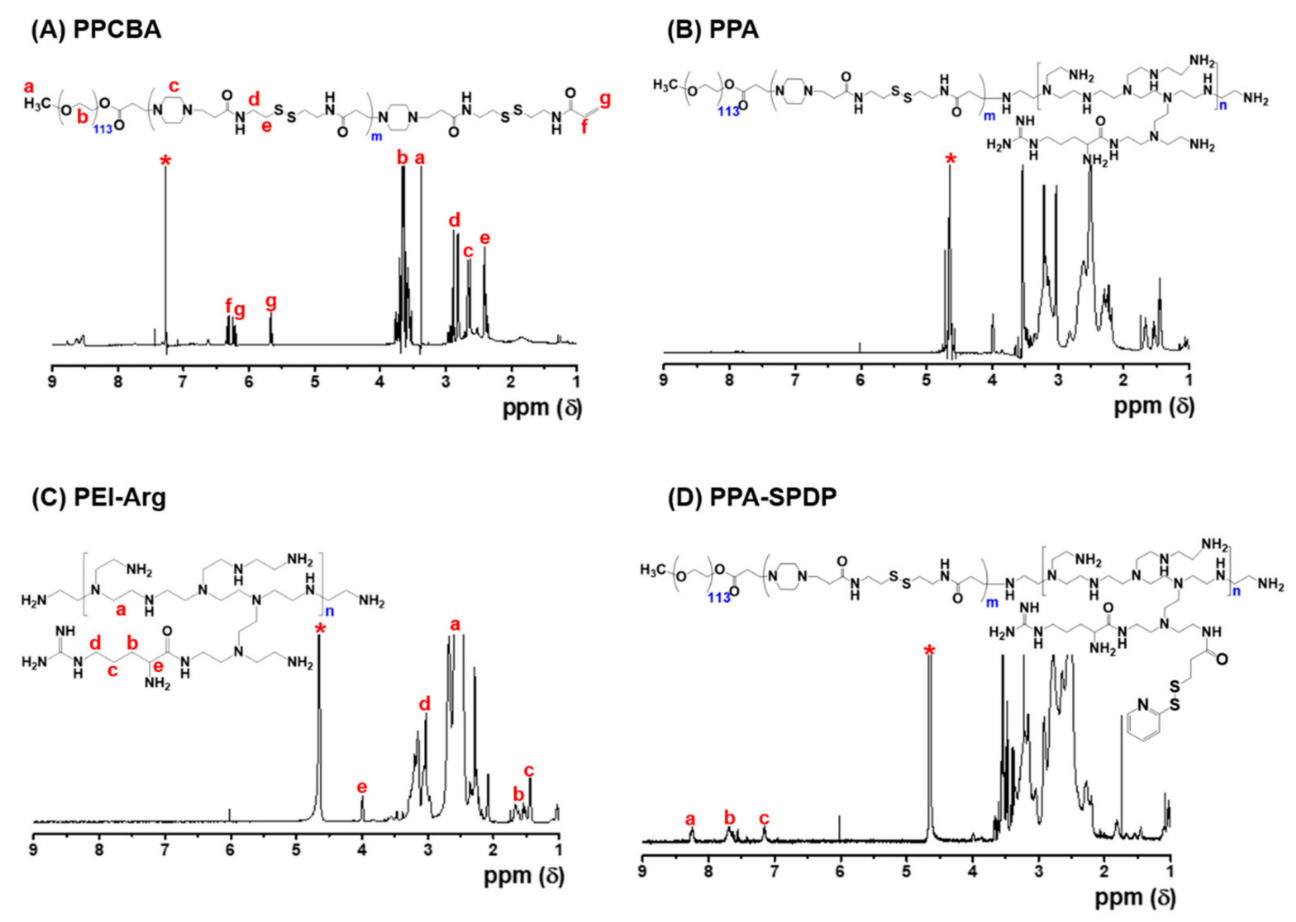
^1^H NMR spectra of (**A**) PPCBA copolymer, (**B**) PEI conjugate variants, (**C**) PPA copolymer, and (**D**) PPA-SPDP copolymer. Asterisks (*) in the spectra denotes residual solvents.

Firstly, the poly(ꞵ-aminoester)-based PPCBA copolymer was prepared by the Michael addition polymerization [46] of mPEG-acrylate and CBA with piperazine. The ^1^H NMR spectrum in Figure 1A shows the presence of PEG, CBA, and piperazine characteristic peaks, which indicated successful preparation of copolymer synthesis. In addition, the copolymers show the presence of acrylate residues at 5.76 and 6.20 ppm. These acrylate residues can be used for postfunctionalization with PEI conjugates. The degree of polymerization (DP) of PCBA block was obtained by comparing the integration of PEG at 3.60 ppm with the –CH_2_– protons of CBA at 2.40 ppm. The DP of PPCBA prepared in this study was found to be 8.08.

Secondly, PEI conjugates with substituents Arg, His, Trp, P1, P2, and P3 were prepared by EDC/NHS chemistry through amide bond formation [47]. The prepared PEI conjugates were confirmed by ^1^H NMR spectrum (Figure 1B). The characteristic peaks of substituents clearly appear in the ^1^H NMR spectra. For PEI-Arg conjugate, the characteristic arginine methylene peaks appeared at 1.46 to 1.80 ppm. On the other hand, PEI-His and PEI-Trp conjugates showed peaks of histidine and tryptophan in the aromatic region along with PEI. These results are in line with the previously reported ^1^H NMR spectra of PEI-Arg, PEI-His and PEI-Trp conjugates, indicating the successful synthesis of PEI conjugates. Similarly, three different piperazine derivatives were conjugated, and the conjugates obtained were subjected to ^1^H NMR analysis. ^1^H NMR spectra of PEI-pip conjugates showed new characteristic peaks, and the peaks were clearly separated beyond 2.50 ppm, indicating successful conjugation. It should be noted that after conjugation with guanidine, the PEI-Gua conjugate showed a single multiplet peak.

Finally, a series of PPA conjugates were synthesized by reacting PEI conjugates with the acrylate end of the PPCBA copolymer. The successful PPA conjugates were confirmed by ^1^H NMR spectra shown in Figure 1C, which showed combined peaks of PEI conjugates and PPCBA copolymers. It should be noted that for all conjugates, the acrylate peaks clearly disappeared, indicating the successful synthesis. All PPA conjugates showed characteristic peaks corresponding to their substituents and the disappearance of acrylate residues.

**Figure 2:**
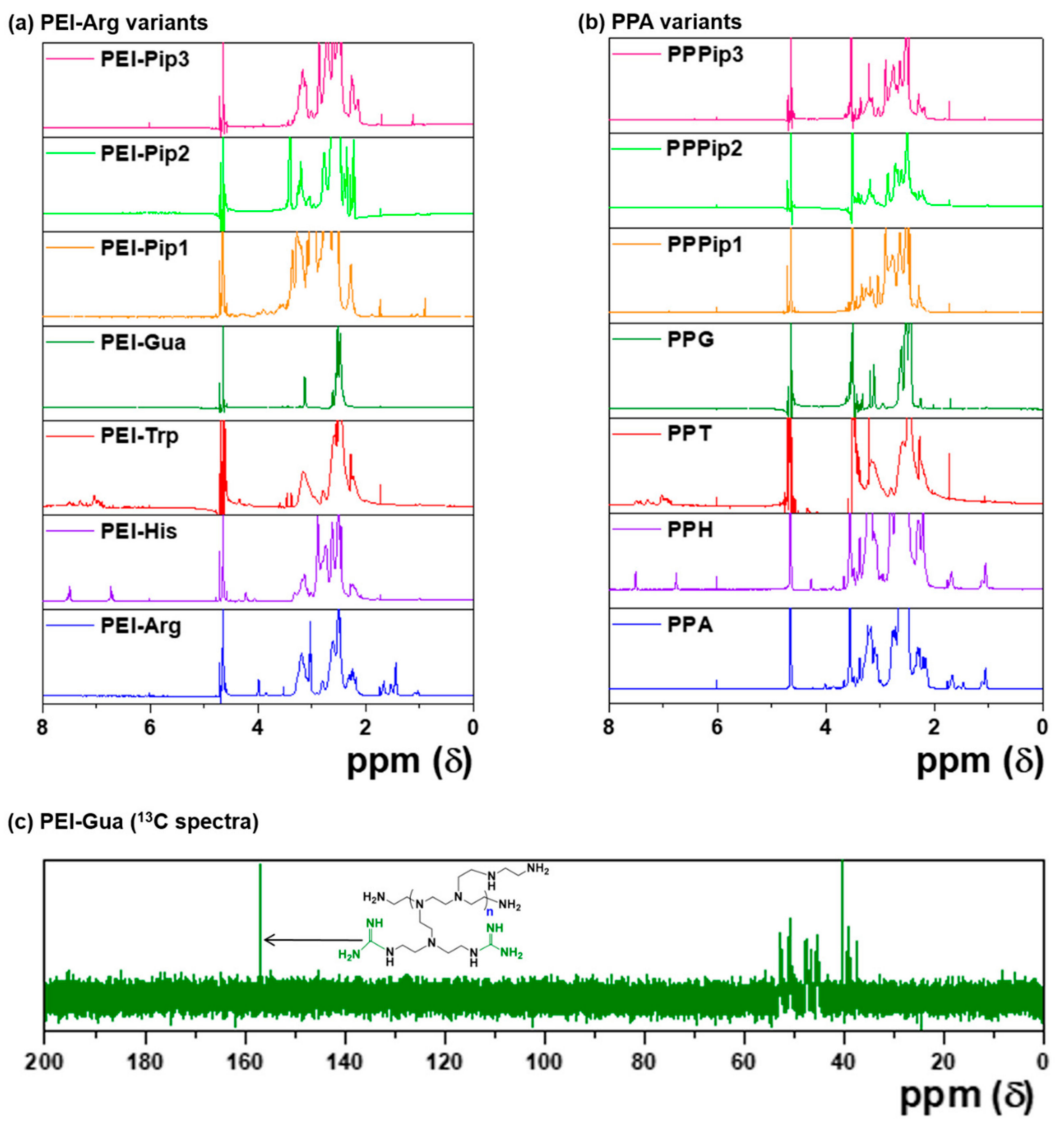
^1^H NMR spectra of (**a**) PEI-Arg variants and (**b**) PPA variants. (**c**) ^13^C NMR spectra of PEI-Gua conjugate.

The PPA copolymers were then reacted with SPDP to obtain PPA-SPDP copolymers for conjugation with Ad. ^1^H NMR spectra of PPA-SPDP in Figure 1D show new characteristic peaks at 7.16, 7.62, and 8.29 ppm corresponding to the pyridyl disulfide residues, indicating successful modification of amine residues in PPA copolymers with SPDP cross-linker. Figure 1 shows the representative ^1^H NMR spectra of PPCBA, PEI-Arg intermediates, and the final PPA copolymer. The ^1^H NMR spectra of all copolymers are summarized in Figure 2. Physicochemical characteristics of PEI conjugates and PPA copolymers are summarized in Table 1.

### 3.2. pH-Buffering Capacity of PPA Copolymers

**Figure 3:**
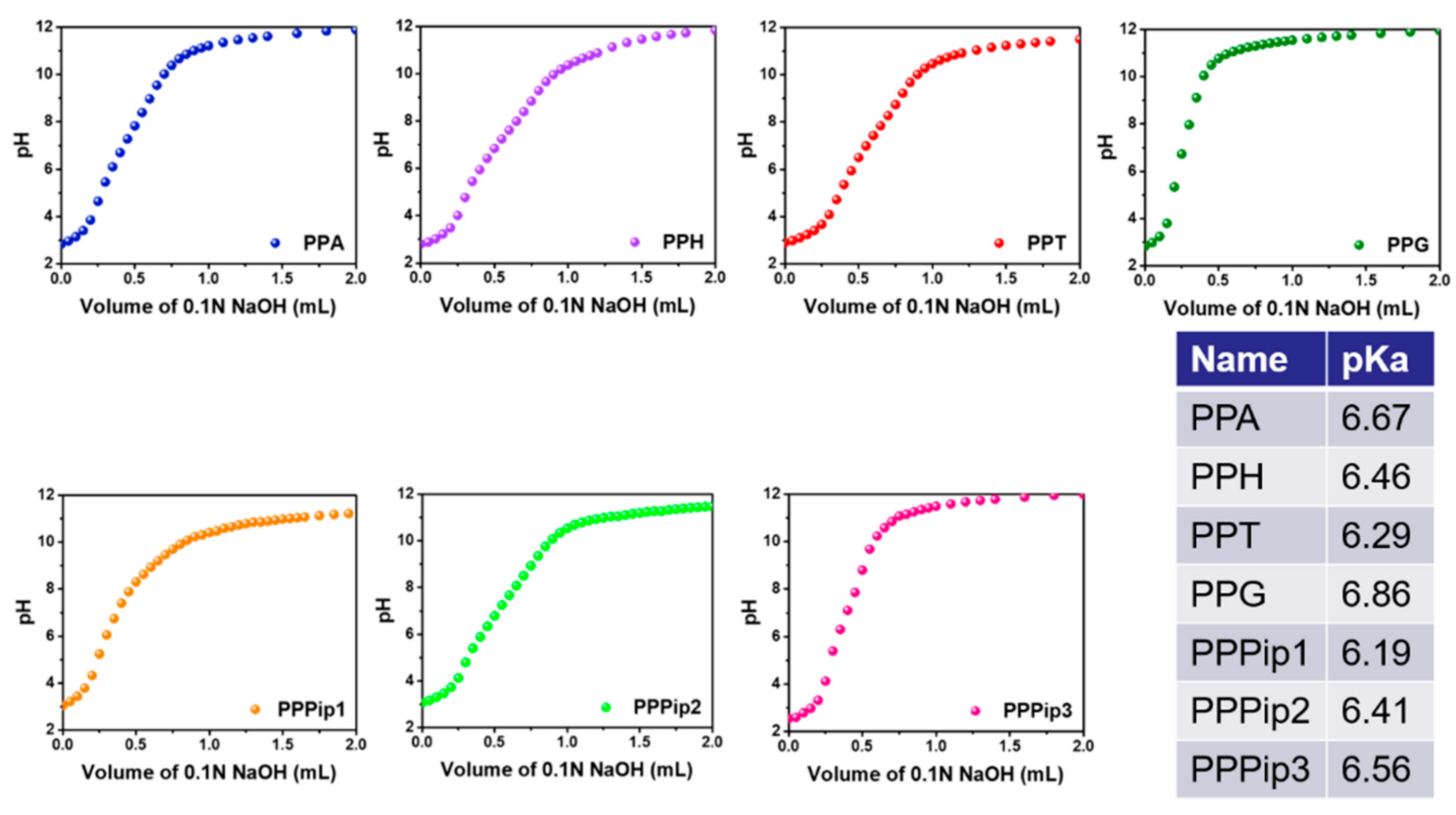
Acid–base titration curve of PPA variants. Inset table is for the corresponding pKa of PPA variants.

It is known that poly(ꞵ-aminoester) [48] and PEI [49] polymers alone or combined in the copolymers can contribute to the pH-buffering capacity in the physiological condition. The pH-buffering capacity of different PPA copolymers was investigated by preparing the conjugate solution in pH 3.0 and assessing the pH-buffering capacity in response to the change in pH of the copolymer solution titrated against 0.1N NaOH. The acid–base titration curves of a series of PPA copolymers are shown in Figure 3. All PPA conjugates exhibited a pH buffering in the range between 4 and 10. From the titration curve, the PPG conjugate exhibited the highest pH-buffering capacity (pH 6.86), which may be due to the concentration of guanidine residues with higher water solubility. In addition, PPA, PPH, and PPPip3 exhibited pH-buffering capacities of 6.67, 6.46, and 6.56, respectively, close to that of PPG conjugates. The other three conjugates, namely PPT, PPPip1, and PPPip2, exhibited pH-buffering capacities of 6.29, 6.19, and 6.41, respectively, which were lower than those of other conjugates. In particular, PPT and PPPip1 showed poor solubility in the basic pH condition. The proton buffering capacity results showed that PPA-based conjugates could escape in the endo/lysosomal pH environment.

In general, the titration point shows a sharp shift at the pH range between 4 and 8 with a lower volume of 0.1N NaOH and then slowly increases until 10 and reaches a plateau. The change in buffering capacity of the conjugates at this point is mainly due to the conjugated substituents in the PEI conjugates. In addition, the presence of poly(ꞵ-amino esters) in the conjugate also contributed to the pH buffering as observed in the previous reports [50]. When the pH was raised above 10, the poly(ꞵ-amino esters) became hydrophobic, and the conjugates form micelles in those pH conditions [51]. At this point, no change in the titration curve was observed. PPA, PPH, PPG, and PPPip3 copolymers with the highest pH-buffering capacity were used for further studies for conjugating with Ad.

### 3.3. Characterization of PPA-SPDP Copolymers

**Figure 4:**
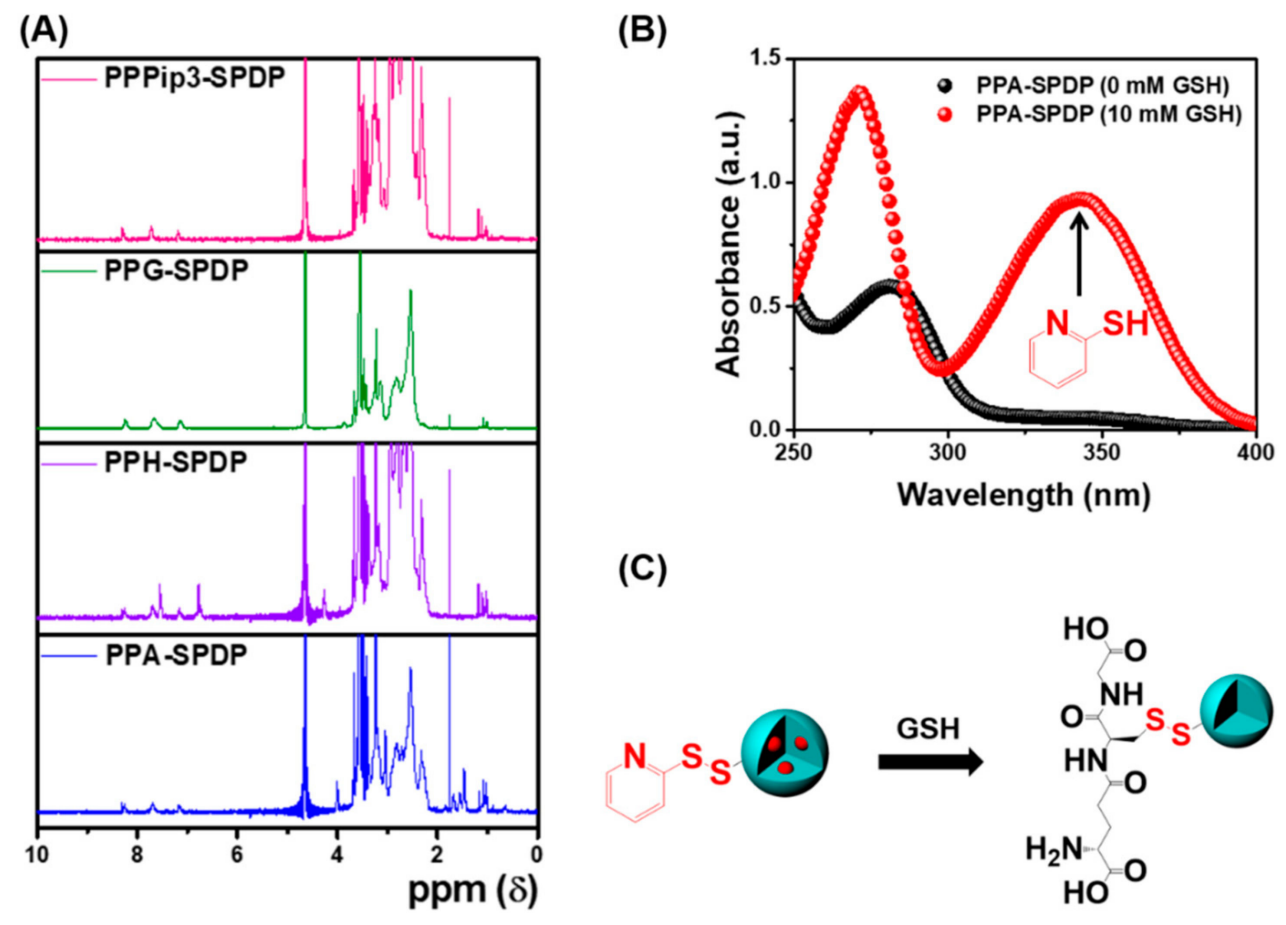
(**A**) ^1^H NMR spectra of PPA-SPDP variants. (**B**) Exposure of PPA-SPDP conjugate with and without GSH and the changes were examined using UV-Vis spectroscopy. (**C**) Schematic illustration of GSH-treated PPA-SPDP conjugate.

The PPA copolymers were coupled with small molecule SPDP for facile conjugation with thiolated Ad (Ad-SH). From the ^1^H NMR spectra (Figure 4A), the characteristic peaks of SPDP appeared at 7.26 to 8.32 ppm, indicating the effective functionalization of SPDP in the PPA conjugates. The cross-linker SPDP containing pyridine and disulfide bonds was used for the facile exchange reaction between sulfhydryl groups and disulfide groups [39]. The functionalization of SPDP cross-linker in the PPA conjugates was confirmed by UV-vis spectroscopy after incubating with GSH (Figure 4B,C). To confirm SPDP functionalization, PPA-SPDP conjugate was incubated with 10 mM GSH, and the change in UV-vis spectrum was measured before and after incubation. Before incubation with GSH, the characteristic peak of pyridine disulfide appeared at 281 nm. Interestingly, after incubation with GSH, a new characteristic peak appeared at 343 nm. This is due to the reduction of disulfide bonds by the active sulfhydryl groups of GSH and release of 2-thiopyridine [52].

**Figure 5:**
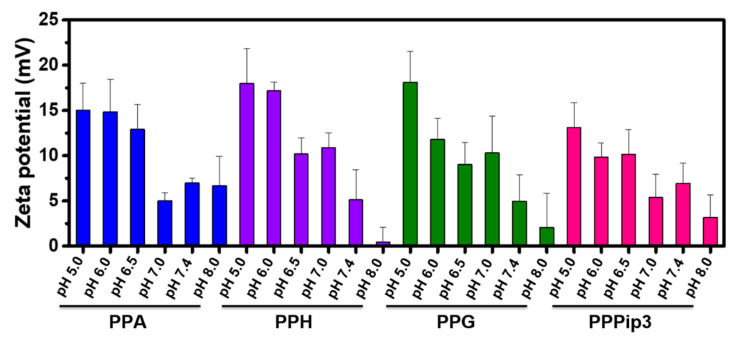
Zeta potential of PPA-SPDP variants at different pHs. For the zeta measurement, 1 mg/mL sample was taken, and the pH was adjusted before measurement. The error bars represent means ± SD of five replicates.

The change in zeta potential of PPA-SPDP copolymers at different pHs was measured to assess the proton buffering. As shown in Figure 5, the zeta potential of the copolymers was gradually increased from 2 to 15 mV when the pH was adjusted from 8.0 to 5.0. Regardless of the copolymers, all the copolymers showed a gradual increase in zeta potential when adjusted from basic to acidic pH conditions. The zeta potential of the copolymers was significantly higher at the endo/lysosomal pH condition, which indicated effective proton buffering capacity at the acidic pH condition [28]. It should be noted that after conjugation of SPDP in the PPA copolymers, the pH-buffering capacity was not affected.

### 3.4. Physiochemical Characterization of PPA-Ad Conjugates

To examine the prepared PPA-SPDP copolymers suitable for conjugation with Ad, PPA-SPDP copolymer was reacted with Ad-SH by simple mixing. Different concentrations of PPA-SPDP copolymers (2 × 10^4^ to 2 × 10^5^) were used for conjugation with a fixed amount of Ad particles (5 × 10^9^ vp), and the size and surface characteristics of the PPA-Ad conjugates were examined. Substituents in the PPA copolymers, including amino acids, piperazines, and guanidine, facilitate interaction with Ad by ionic interaction, and the presence of SPDP in the conjugates forms a stable covalent bond between PPA and Ad. The presence of pH-responsive residues facilitates ionic interaction with negatively charged surface cell membranes. In particular, the presence of histidine and piperazines in the conjugates provides fusogenic properties when they are exposed to acidic pH conditions [28]. In addition, such conjugates show enhanced gene transfer efficiency and reduced toxicity.

**Figure 6:**
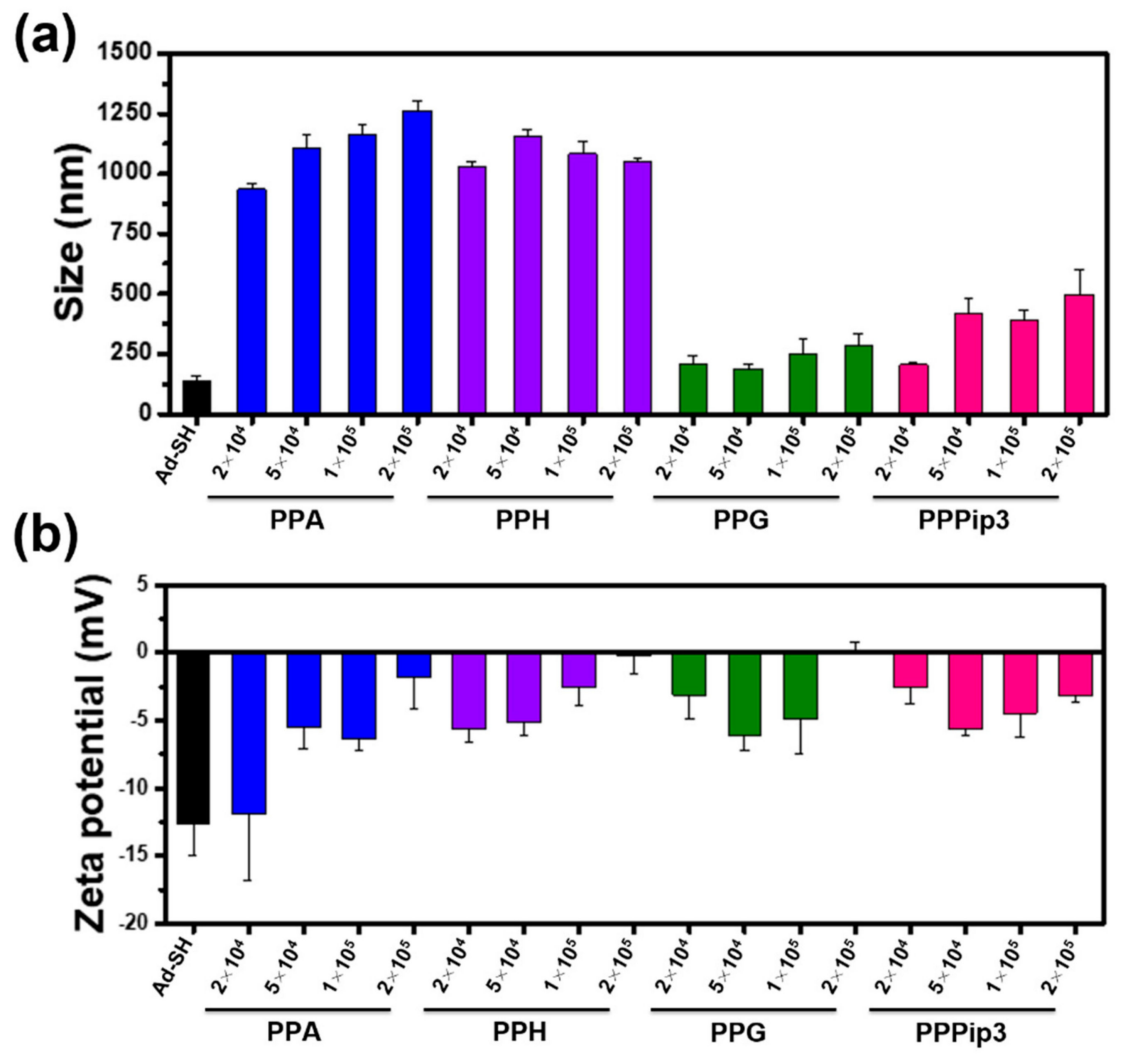
(**a**) Average size and (**b**) zeta potential of PAA-SPDP variants conjugated with Ad-SH. For conjugation, fixed amount of Ad-SH (5 × 10^9^ vp) was reacted with various molar ratios of PPA-SPDP (2 × 10^4^, 5 × 10^4^, 1 × 10^5^, or 2 × 10^5^) at pH 7.4. The error bars represent means ± SD of five replicates.

It is known that the size and surface zeta potential of a PPA-Ad nanocomplex affect the transduction efficacy due to the presence of various in vivo factors, including reticuloendothelial system clearance. The negatively charged Ad was converted to neutral or positive after conjugating with PPA copolymers, which indicated that cationic nanoparticles covered the Ad capsids effectively (Figure 6A). The size of PPA copolymers increased as the feed amount of PPA to Ad increased (Figure 6B). The particle size was high due to the protonated form, and H^+^ concentration showed a swollen structure due to the repulsion with the network. On the other hand, PPG and PPPip3 conjugates formed a compact structure by the combination of covalent bonding and ionic interaction between Ad capsids and copolymer residues.

**Figure 7:**
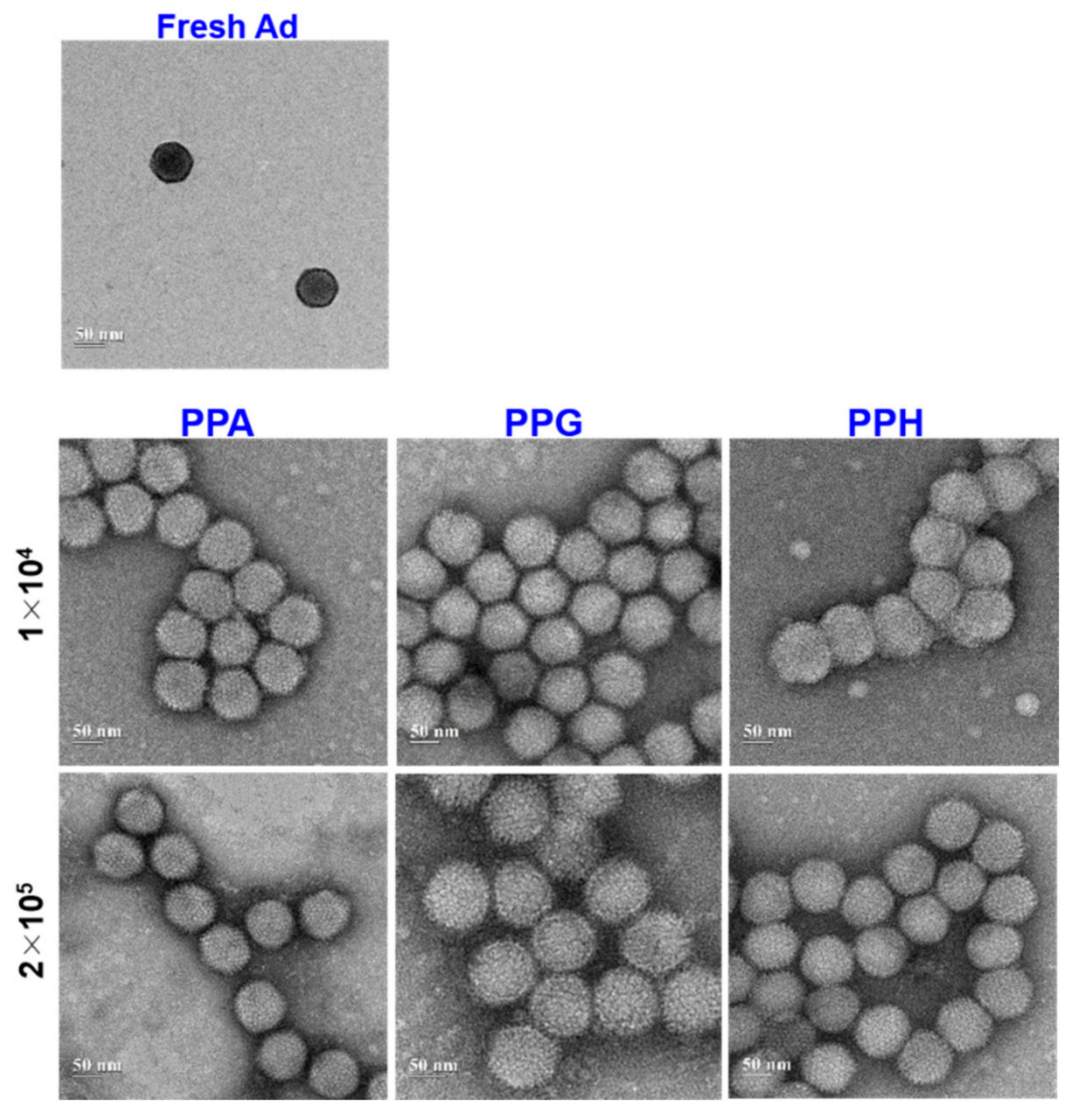
TEM images of fresh Ad and PPA-Ad, PPH-Ad, and PPG-Ad conjugates prepared at two different molar ratios (1 × 10^4^ and 2 × 10^5^) at pH 7.4.

The conjugation efficiency and surface covering of the Ad capsids were examined by TEM. Photomicrographs of free Ad and PPA-Ad conjugates are shown in Figure 7. As expected, free Ad exhibits a hexon structure with an icosahedral shape, a major characteristic of Ad viral particles. This was in agreement with our previously reported naked Ad particles [30]. On the other hand, the Ad particles were covered and exhibited a series of clusters after conjugating with PPA copolymers. In particular, the clusters were evenly separated at a high molar ratio of copolymers to Ad particles. TEM imaging results demonstrated that PPA-SPDP copolymers were effectively conjugated and covered the surface of Ad capsids. Such covering is important during transduction of Ad, as the cationic surface of PPA-Ad conjugates allows the effective internalization of viral particles to cancer cells [53].

### 3.5. Gel Retardation Assay

**Figure 8:**
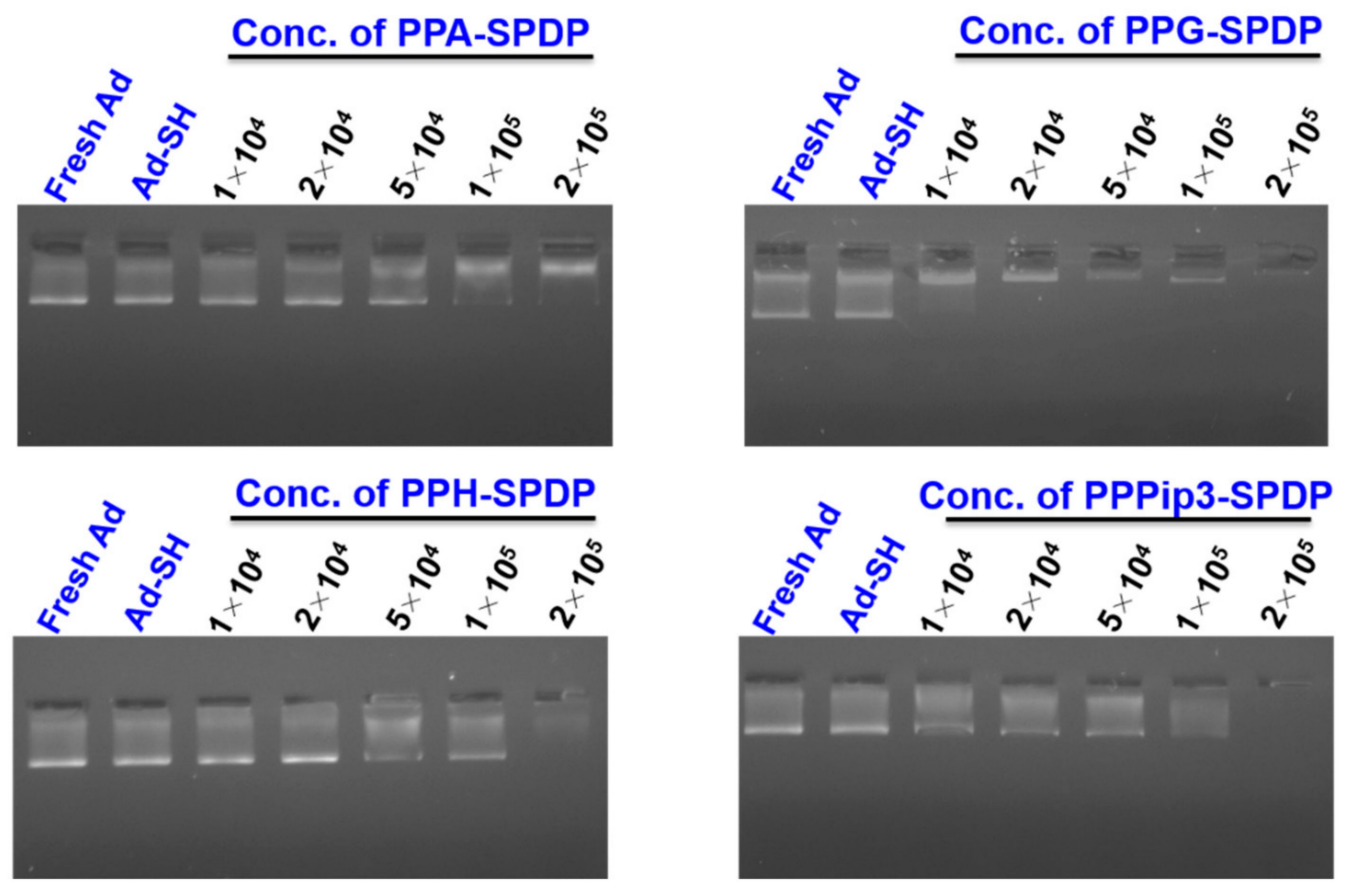
Gel retardation assay of PPA-Ad, PPH-Ad, PPG-Ad, and PPPip3-Ad conjugates prepared at various molar ratios of copolymers (1 × 10^4^, 2 × 10^4^, 5 × 10^4^, 1 × 10^5^, or 2 × 10^5^). Fresh Ad and Ad-SH were also used to confirm the effective covering of Ad capsids.

For the successful transduction of Ad particles, in vitro stability and integrity of the PPA-Ad conjugates are necessary [54]. The effective shielding of Ad by PPA copolymers was confirmed using gel retardation assay (Figure 8 and Table 2). To find the optimal PPA copolymer concentration for covering Ad particles, five different concentrations of copolymers (1 × 10^4^, 2 × 10^4^, 5 × 10^4^, 1 × 10^5^, or 2 × 10^5^) were used with Ad particles (5 × 10^9^ vp). From the gel retardation test, it was found that all four copolymers could effectively inhibit the migration of Ad particles, which indicated the effective conjugation of copolymers to Ad. Regardless of the copolymers, all the copolymers showed protection from lysis buffer at 5 × 10^4^ copolymer concentration and retarded the Ad migration. At higher copolymer concentrations, complete retardation of the Ad particles was observed because of non-migration of the gel from the well.

### 3.6. Cytotoxicity Test of PPA Copolymers

**Figure 9:**
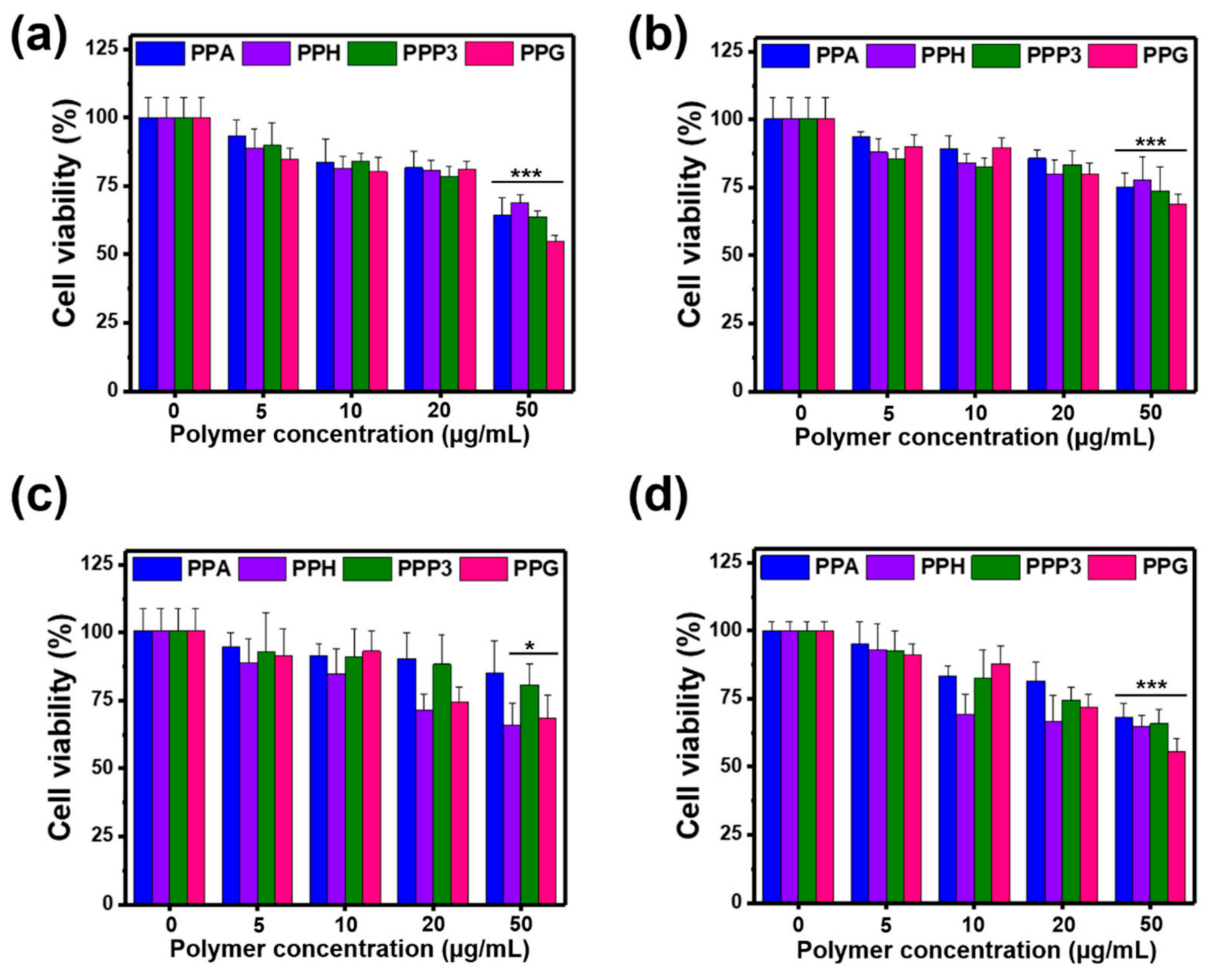
In vitro cell viability of PPA, PPH, PPG, and PPPip3 copolymers on (**a**) A549 cells, (**b**) H460 cells, (**c**) MDA-MB-231 cells, and (**d**) T24 cells. For measurement, cells were exposed to different concentrations of copolymer for 72 h, and the cells were subjected to MTT analysis. The error bars represent means ± SD (*n* = 3). *** *p* < 0.001 and * *p* < 0.01 compared with control (0 µg/mL).

To investigate the nontoxic property of PPA copolymers, MTT assays were performed on A549 cells, H460 cells, MDA-MB-231 cells, and T24 cells treated with PPA variants (Figure 9).

Cells were exposed to different concentrations of copolymer, from 0.5 to 10 μg/mL, for 72 h. No toxic effects were observed on PPA variants, up to 10 μg/mL. Upon exposure to 20 μg/mL of PPA variants, except PPH copolymer, the cell viability of all the cells was above 80%, which indicated the biocompatibility of the PPA conjugates. These results are consistent with previous reports demonstrating that poly(ꞵ-amino esters)-based copolymers exhibit no apparent toxicity to mammalian cells [55]. It is noteworthy that PEI copolymers often show toxicity even at low concentrations [56], and in this study, the toxicity of low PEI molecular weight (1.8 kDa) was effectively reduced after suitable modification with amino acids and piperazines. The presence of PPCBA in copolymers was also played an important role in reducing the toxicity.

### 3.7. Transduction Efficiency of PPA-Ad Complex

**Figure 10:**
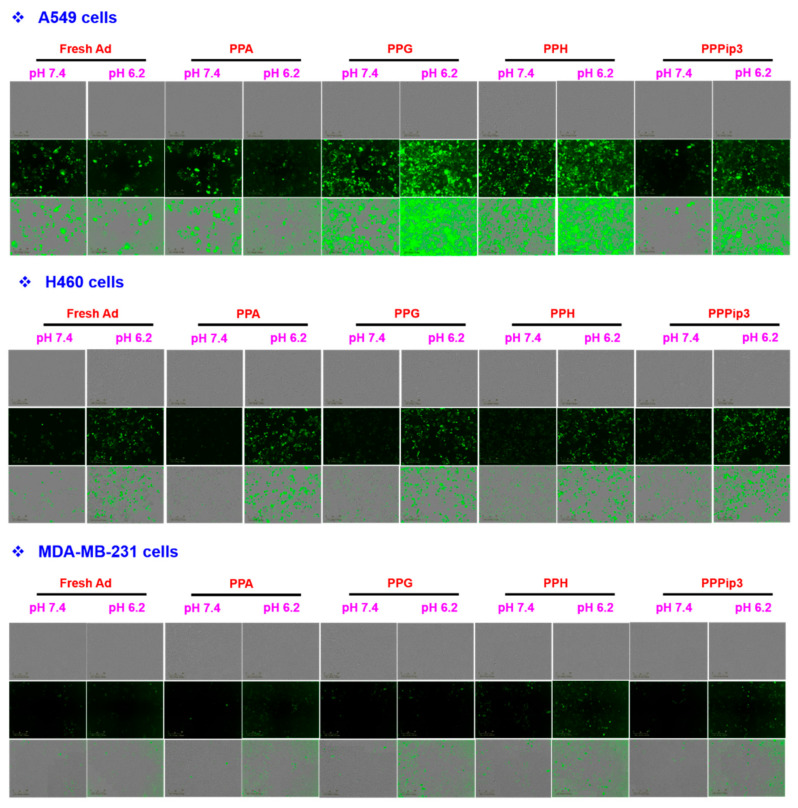

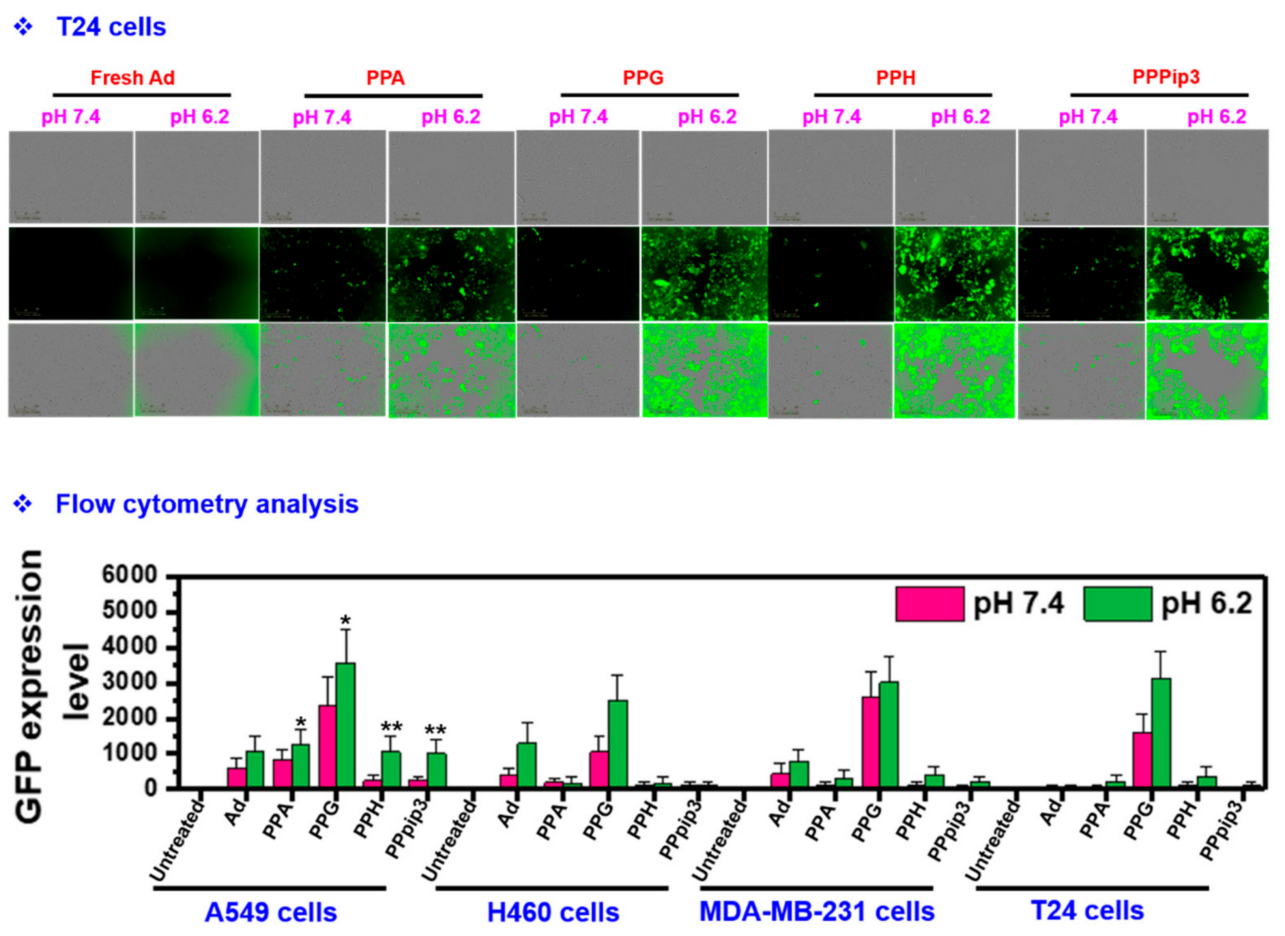
Transduction efficiency of PPA-dE1/GFP-Ad, PPH-dE1/GFP-Ad, PPG-dE1/GFP-Ad, and PPPip3-dE1/GFP-Ad conjugates in different pH conditions (pH 7.4 and pH 6.2). Fluorescence images of CAR-positive and CAR-negative cancer cells exposed to different copolymer–Ad conjugates prepared using copolymer ratio of 5 × 10^4^ and 20 MOI dE1/GFP-Ad. To observe the transduction, cells were exposed to copolymer–Ad conjugates for 60 min, subsequently washed, and incubated for 48 h. The GFP images were photographed using Incucyte Live-Cell imaging system. GFP expression levels were measured by flow cytometry analysis. The error bars in the graph represent standard deviation (*n* = 3). ** *p* < 0.005 and * *p* < 0.01 compared with Ad.

**Figure 11:**
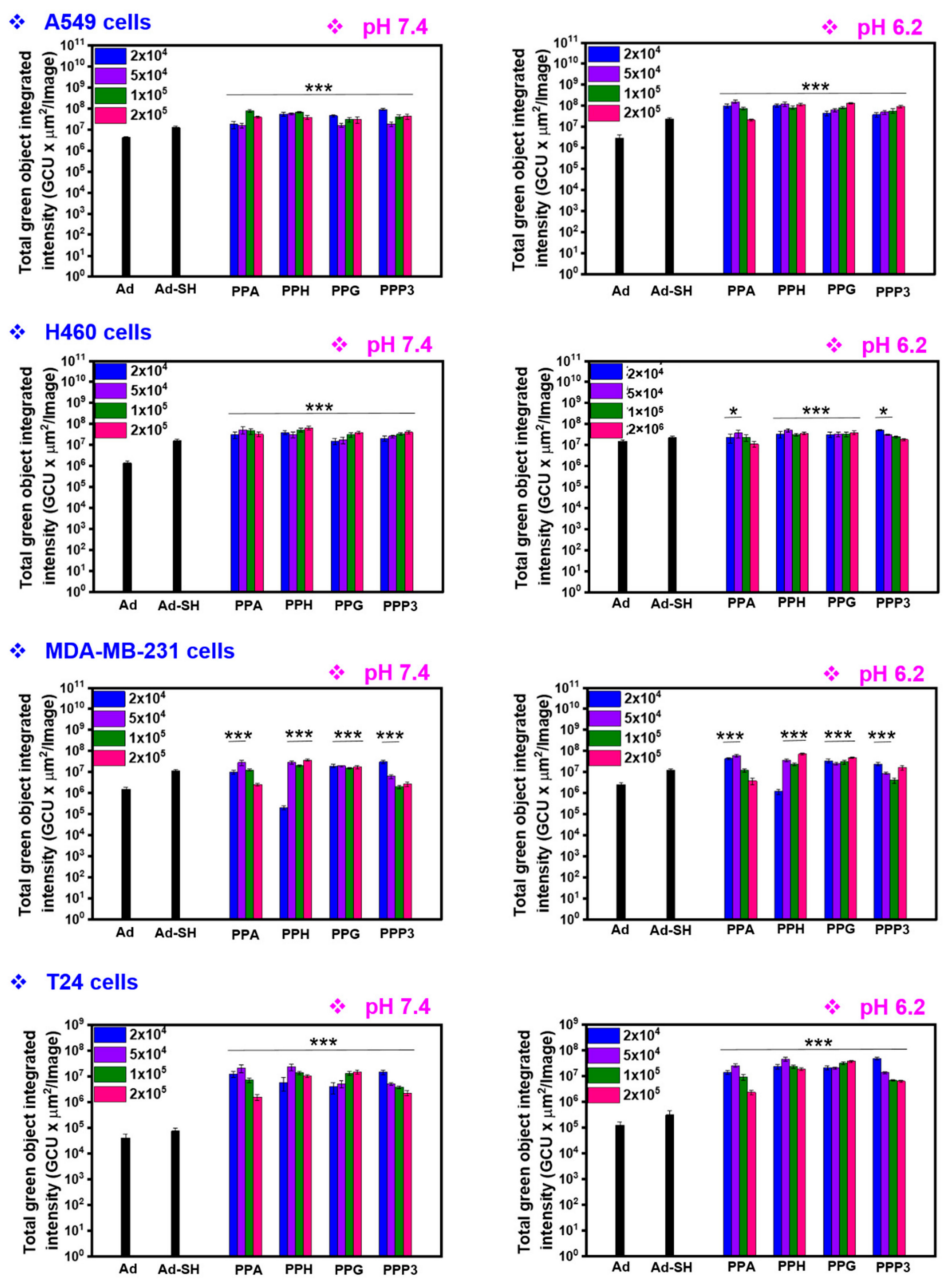
Quantitative analysis of GFP expression from Figure 10. The error bars represent means ± SD (*n* = 3). *** *p* < 0.001 and * *p* < 0.01 compared with Ad.

CAR expression on the surface of cancer cells is an important factor for effective internalization of Ad and subsequent gene expression for improved gene therapy [57]. However, cancer cells often have either no or low levels of CAR expression, which resulted in poor efficacy of Ads in clinics [14]. We previously demonstrated that complexation or mixing of cationic polymers with Ad capsid improved the charge-mediated internalization of the Ad and improved the uptake of Ad into cancer cells even in cancer cells with low levels of CAR expression [31,42,57]. Generally, the cationic polymer has a positive surface charge and can effectively form a complex with negatively charged genetic materials, including Ad [58]. Therefore, we examined the transduction efficiency of PPA-Ad conjugate in various cancer cells with different levels of CAR expression. Both CAR-positive (A549 and H460 cells) and CAR-negative (MDA-MB-231 and T24 cells) were transduced with the same viral dose of fresh Ad or PPA-Ad conjugates with a low molar ratio. Interestingly, as the concentration of PPA copolymer increased, the transduction efficiency significantly increased in acidic pH condition (pH 6.2), indicating the pH-sensitive property of the copolymer. Fluorescence microscopic images showed copolymer-concentration-dependent increase in transduction efficacy by PPA-Ad complex with increasing molar ratio of PPA in both CAR-positive and CAR-negative cells when compared to naked Ad (Figure 10). Importantly, transduction with PPA-Ad conjugates formed at a copolymer:Ad molar ratio of 5 × 10^4^ yielded the highest GFP expression level, which was higher than that achieved with fresh Ad in CAR-positive cells (Figure 11). In addition, PPA-Ad conjugate (polymer:Ad molar ratio of 5 × 10^4^) showed higher transduction efficiency in CAR-negative MDA-MB-231 and T24 cells compared to naked Ad, which indicated that PPA-Ad conjugate is internalized independently and overcomes CAR-dependent cell entry. Together, these observations demonstrate that PPA-Ad conjugate can overcome the CAR-dependent internalization of Ad and improve the delivery of Ad in CAR-positive and CAR-negative cancer cells.

## 4. Conclusions

In summary, a series of pH- and bioreducible PPA copolymers were synthesized and conjugated with Ad for the delivery of therapeutic genes to cancer cells. The PPA copolymers functionalized with SPDP cross-linker allow the effective conjugation with Ad. Such conjugation converts the negatively charged Ad to a positively charged nanocomplex for effective ionic interaction with cancer cells. The PPA copolymers were found to be nontoxic to various cancer cells. The PPA-Ad conjugates showed increased transduction efficiency in both CAR-positive and CAR-negative cancer cells, and such maneuvered complex improves the potential utility of Ad vectors in the clinical setting. These results suggested the potential of pH- and bioreducible PPA copolymers as a suitable nonviral vector for fabricating an advanced Ad system for cancer gene therapy.

## Figures and Tables

**Table 1:** Physicochemical characterization of PPA copolymers.

Library	PPCBA (Degree of Polymerization)	Acrylate Consumption (%)	bPEI Substitution Moieties	Degree of Substitution (Each Moieties) ^a^	Degree of Substitution of SPDP ^a^
PPA	8.08	100	Arginine	5.03	2.36
PPH	8.08	100	Histidine	4.93	1.98
PPT	8.08	100	Tryptophan	4.72	1.71
PPG	8.08	100	Guanidine	3.86	1.78
PPPip1	8.08	100	Piperazine	4.66	2.61
PPPip2	8.08	100	Piperazine	3.64	2.05
PPPip3	8.08	100	Piperazine	4.54	2.72

^a^ Calculated using ^1^H NMR spectra.

**Table 2:** With increasing polymer concentration. Gel photographs were analyzed using ImageJ software. The optical density was normalized by naked Ad in each group.

	Ad	Ad-SH	1 × 10^4^	2 × 10^4^	5 × 10^4^	1 × 10^5^	2 × 10^5^
Ad	5209.719	4537.426	4419.841	3289.477	2776.355	231.556	91.435
Ad	2851.991	2762.406	2694.941	1672.941	328.385	*	*
Ad	2762.820	2690.234	*	*	*	*	*
Ad	3706.184	3220.062	622.335	435.385	46.192	*	*

(* Asterisks denotes no bands or bands with poor intensity).

## Data Availability

The data that support the findings of this study are available from the corresponding authors, upon reasonable request.
